# Supplementary material for: Chronic Overexpression of Neuronal NRG1-III in Mice Causes Long-Term Detrimental Changes in Lower Motor Neurons, Neuromuscular Synapses and Motor Behaviour
Source: Int J Mol Sci. 2025 Nov 26;26(23):11421. doi: 10.3390/ijms262311421 (PMC12692416; doi:10.3390/ijms262311421)
Supplement: Supplementary file 1 [file ijms-26-11421-s001.zip › SUPPLEMENTARY Figure Legends ijms3974779_121125.pdf]

SUPPLEMENTARY FIGURE S1. Motor performance analyses using the Catwalk XT analysis system in WT and TG mice. The paw intensity of the front and hind limbs (both right and left) was examined in middle-aged and old mice at different time points. Reduced paw intensity is observed at several time points with significant differences in some of them. Simple sizes n= 8-24 per time point (one WT point at n= 2 and some WT points at n= 4). *p < 0.05, **p < 0.01 (Student's *t*-test for genotype comparisons).

SUPPLEMENTARY FIGURE S2. Representative micrographs of entire L4 VRs semithin sections (a detailed view of Figure 1C). Scale bar= 100 μm.

SUPPLEMENTARY FIGURE S3. NRG1-III overexpression in cultured MNs alters the expression and localization of other C-bouton-associated postsynaptic proteins without promoting the formation of cholinergic VAChT-positive afferent synapses on MNs. A-D) Triple immunostaining for ChAT together with some C-bouton and synaptic markers. **A)** In TG MNs, NRG1 and Kv2.1 exhibit an expanded peripheral distribution mimicking the *in vivo* pattern, whereas WT MNs display the typical spot-like pattern at the periphery (arrows). **B)** The M2 muscarinic acetylcholine receptor is increased at the surface of TG MNs; however, M2 does not colocalize with the synaptic marker SYN in WT and does not seem to be specifically associated with SYN in TG (arrows). **C)** In WT MNs, NRG1 labeling at the periphery does not overlap with SYN. In the panel, one TG MNs showing a marked expansion of NRG1, exhibit an extensive coverage of SYN-positive boutons similar to that observed in WT. **D)** VAChT-positive synaptic boutons showing the infrequent colocalization with NRG1, as depicted in one WT MN (arrows), but not in another within the same field. Examples of TG MNs in which no colocalization between VAChT and NRG1 is observed (arrows). Scale bar= 10 μm.

SUPPLEMENTARY FIGURE S4. TG MNs *in vitro* show reduced vulnerability to glutamate-mediated excitotoxicity and decreased expression of glutamate receptors. A) MNs are visualized by ChAT (green) and DAPI (blue) fluorescent labeling after 17 DIV in control and after stimulation with 100 μM N-Methyl-D-aspartate acid (NMDA) for 30 min. **B)** Quantification of MN density after

NMDA. **C)** Western blot of the glutamate receptors GluN2A, GluN2B, and GluN1 showing a trend toward reduction in TG cultures in relation to WT (17 DIV). **D)** GluN2B and GluA2 (green) and ChAT (red) immunoreactivity combined with DAPI (blue) (17 DIV). Note the accumulation of both markers on MN surface (arrows). **E, F)** Quantification revealing lower GluN2B levels in TG MNs while GluA2 expression remains unchanged compared to WT. **G)** Cytoplasmic-RE Ca^{2+} dynamics following ER Ca^{2+} replenishment and subsequent stimulation with caffeine and thapsigargin. **H)** Ca^{2+} imaging in MNs from WT and TG (15-19 DIV), showing no differences in intracellular Ca^{2+} transients following stimulation with NMDA, kainic acid (KA) or KCl. Sample sizes: MN density, n= 60 images from 6 cultures; western blot, n= 4-8 animals; GluN2B and GluA2 immunoreactivity, n= 22-24 MNs from 2 cultures; Ca^{2+} levels after NMDA, KA, and KCl stimulation, n= 3 experiments, 46-118 MNs; Ca^{2+} levels after KCl, caffeine, and thapsigargin stimulation, n= 4 experiments, 39-218 MNs (two-way ANOVA, Bonferroni‘s *post hoc* test). **p < 0.01 and ****p < 0.0001. Scale bars: 100 µm in **(A)** and 10 µm in **(D)**.
