# Supplementary figures and images for: Chronic Overexpression of Neuronal NRG1-III in Mice Causes Long-Term Detrimental Changes in Lower Motor Neurons, Neuromuscular Synapses and Motor Behaviour
Source: Int J Mol Sci. 2025 Nov 26;26(23):11421. doi: 10.3390/ijms262311421 (PMC12692416; doi:10.3390/ijms262311421)

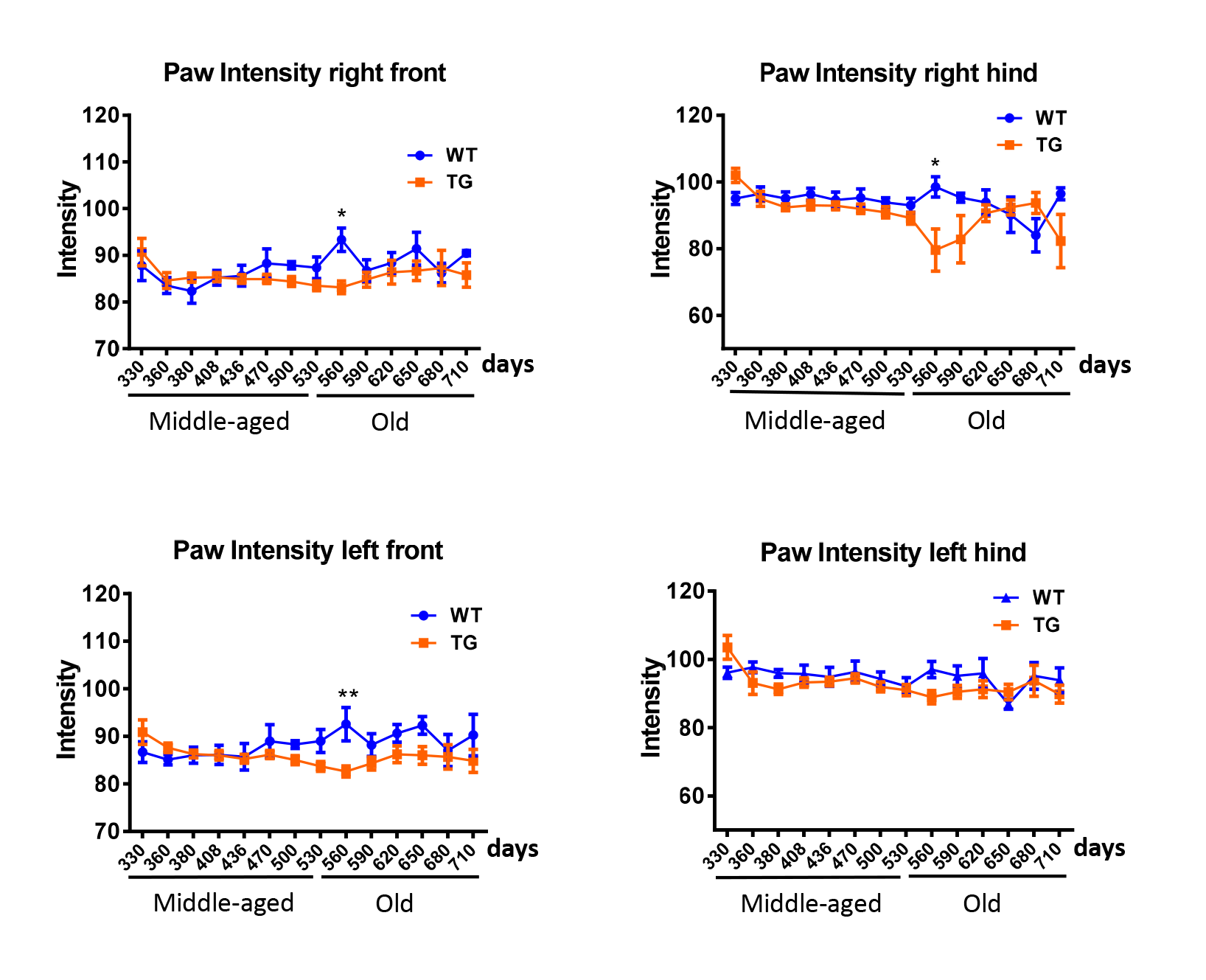

Supplement: Supplementary file 1 [file ijms-26-11421-s001.zip › Supplementary Figure S1.tif]

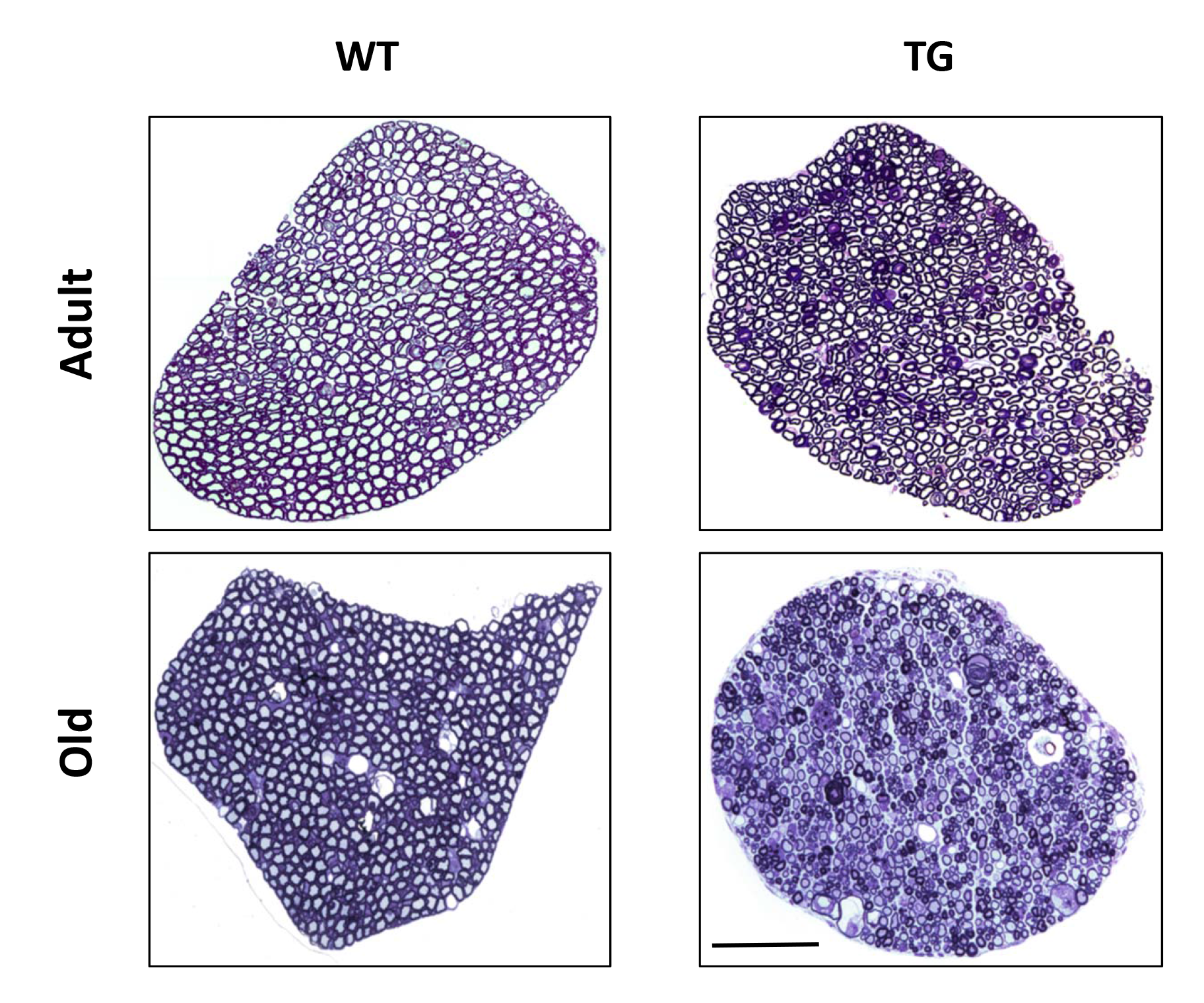

Supplement: Supplementary file 1 [file ijms-26-11421-s001.zip › Supplementary Figure S2.tif]

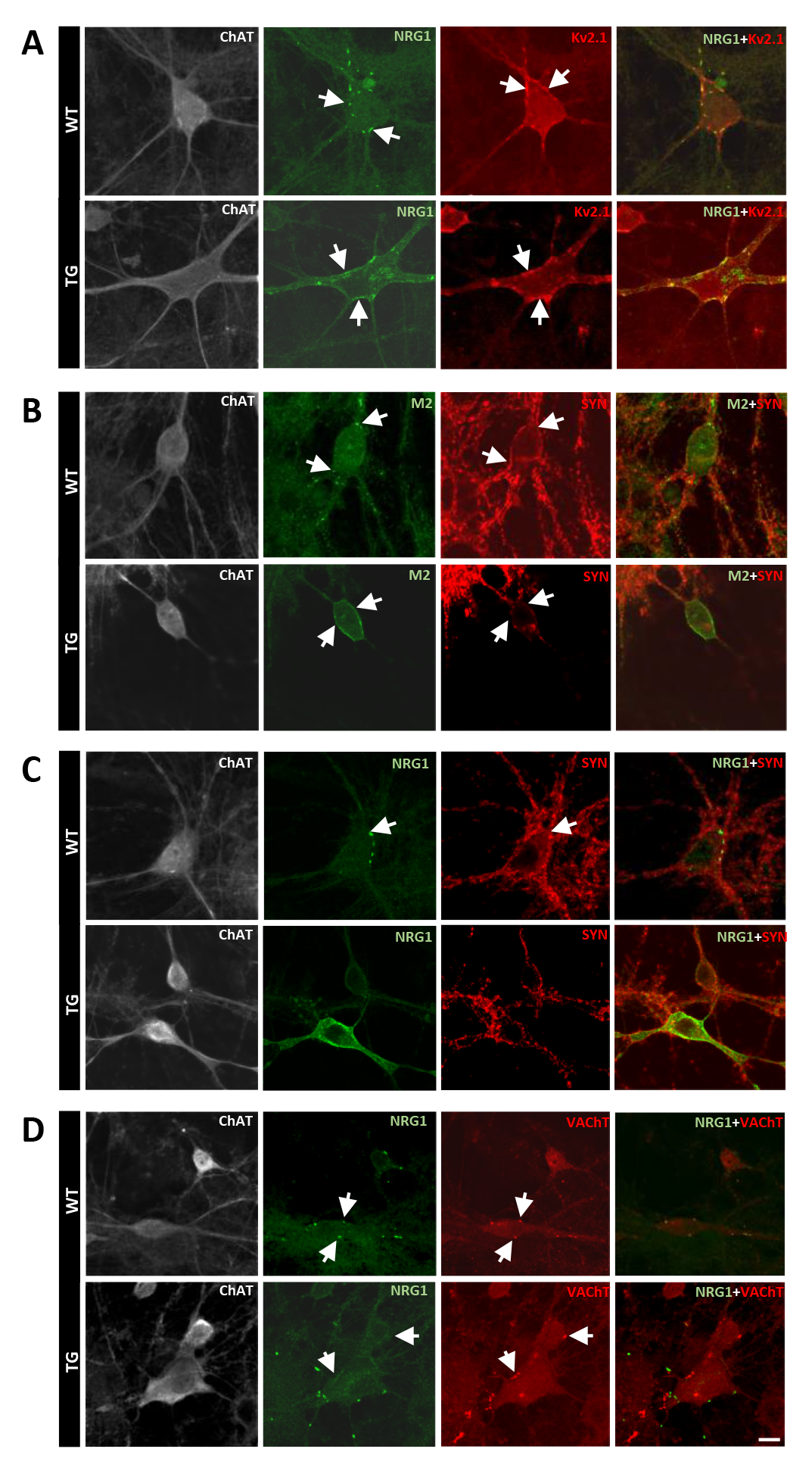

Supplement: Supplementary file 1 [file ijms-26-11421-s001.zip › Supplementary Figure S3.tif]

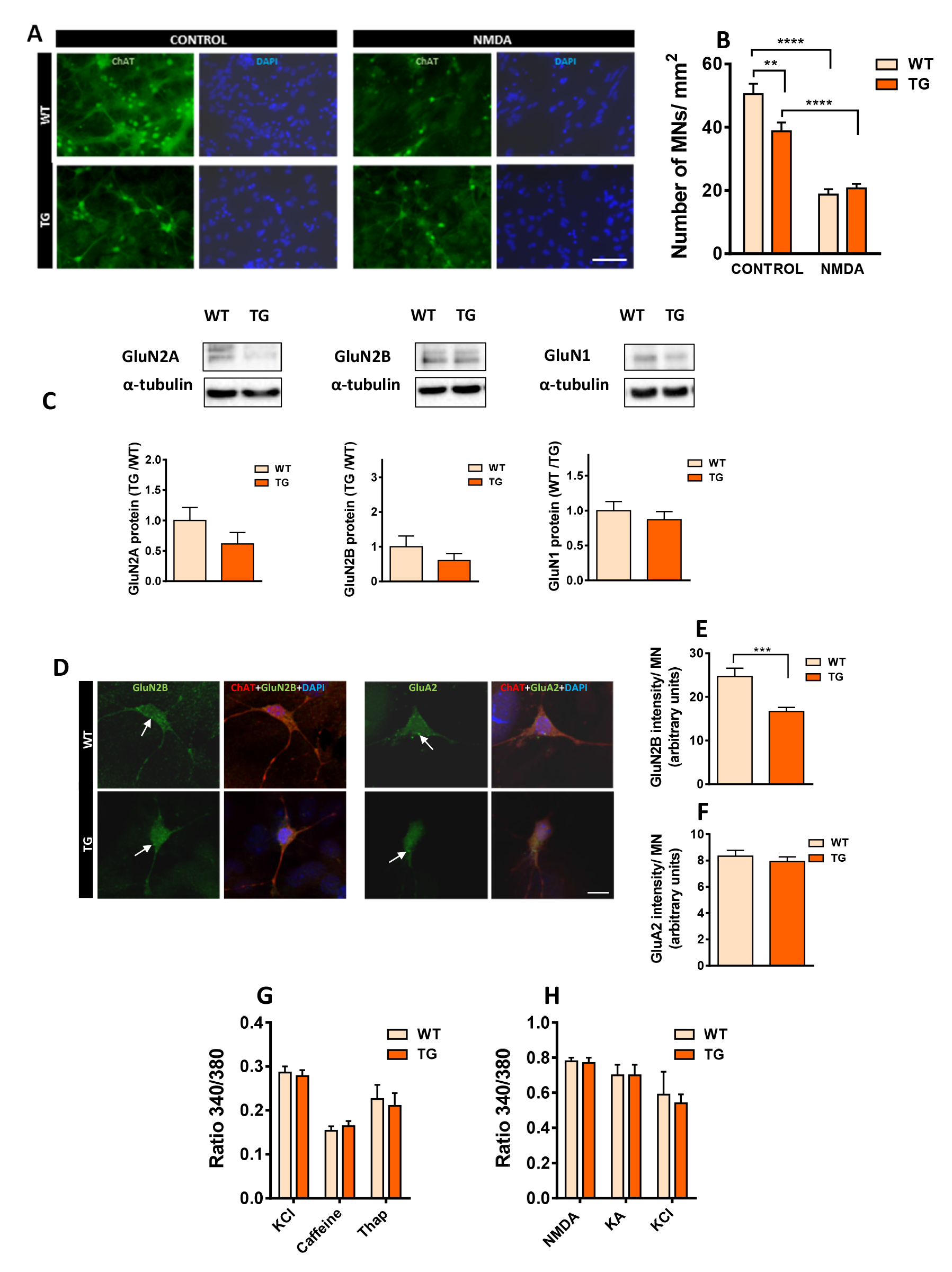

Supplement: Supplementary file 1 [file ijms-26-11421-s001.zip › supplementary Figure S4.tif]
